# SIMILARITY IN HEALTH-RELATED BEHAVIORS WITHIN OLDER ADULTS’ CLOSE SOCIAL RELATIONSHIPS

**DOI:** 10.1093/geroni/igad104.0495

**Published:** 2023-12-21

**Authors:** Noah Webster, Kristi Chin

**Affiliations:** University of Michigan, Ann Arbor, Michigan, United States; University of Michigan, Ann Arbor, Michigan, United States

## Abstract

Health-related behaviors are socially contagious, spreading through social networks via multiple mechanisms. It remains unclear though which mechanisms are most salient among older adults. Guided by Social Contagion Theory and the Convoy Model of Social Relations we examine similarity in health-related behaviors (exercise, smoking, alcohol consumption) between older adults and their closest social network members and investigate associations with multiple social factors (homophily, network structure, relationship quality). Data are from Wave 3 of the Detroit-based Social Relations Study collected in 2015. Panel respondents nominated people who were close and important in their lives, and up to the first three nominated were interviewed. Analyses were conducted at the dyad-level, and we selected dyads in which at least one member was age 60 or older (n=247 dyads). Generalized estimating equations were used due to some panel respondents being in multiple dyads. Greater homophily was found to be associated with similarity in both exercise frequency and number of alcoholic drinks consumed per week. Specifically, pairs of close social network members who were more similar in self-rated health were significantly more similar in exercise frequency, and those of the same gender and more similar in age were significantly more similar in alcohol consumption. Network structure, specifically greater contact frequency, was associated with significantly more similarity in alcohol consumption, but less similarity in exercise frequency. Findings suggest the salience of multiple aspects of older adults’ close social relationships that can be incorporated into behavior change interventions and leveraged to encourage healthy lifestyles in later life.

